# The Mutational Landscape of Pancreatic and Liver Cancers, as Represented by Circulating Tumor DNA

**DOI:** 10.3389/fonc.2019.00952

**Published:** 2019-09-24

**Authors:** Alistair Rice, Armando del Rio Hernandez

**Affiliations:** Cellular and Molecular Biomechanics Laboratory, Department of Bioengineering, Faculty of Engineering, Imperial College London, South Kensington Campus, London, United Kingdom

**Keywords:** circulating tumor DNA (ctDNA), pancreatic ductal adenocarcinoma (PDAC), hepatocellular cancer (HCC), somatic mutations in cancer, tumor heterogeneity

## Abstract

The mutational landscapes of pancreatic and liver cancers share many common genetic alterations which drive cancer progression. However, these mutations do not occur in all cases of these diseases, and this tumoral heterogeneity impedes diagnosis, prognosis, and therapeutic development. One minimally invasive method for the evaluation of tumor mutations is the analysis of circulating tumor DNA (ctDNA), released through apoptosis, necrosis, and active secretion by tumor cells into various body fluids. By observing mutations in those genes which promote transformation by controlling the cell cycle and oncogenic signaling pathways, a representation of the mutational profile of the tumor is revealed. The analysis of ctDNA is a promising technique for investigating these two gastrointestinal cancers, as many studies have reported on the accuracy of ctDNA assessment for diagnosis and prognosis using a variety of techniques.

## Introduction

Both pancreatic and liver cancers show high mortality rates and a poor outcome, in part due to a complex and heterogeneous mutational landscape that hinders diagnosis and prognosis. The detection of this mutational profile has traditionally required tissue biopsy, a highly invasive procedure. Recent developments have indicated the potential of liquid biopsies, such as those which analyse circulating tumor DNA (ctDNA) (1). By understanding the mutational landscape of these tumors, and to what extent the ctDNA mutational landscape reflects this, our understanding of how liquid biopsies can be useful in personalized therapy will be improved.

Pancreatic and liver cancer most commonly present in the forms of pancreatic ductal adenocarcinoma (PDAC) and hepatocellular carcinoma (HCC). PDAC is the fourth most common cause of cancer death and thirteenth most common cancer, with incidence and mortality on the increase. Risk factors include chronic pancreatitis, alcohol abuse and obesity (2). HCC is the second leading cause of cancer death worldwide and the sixth most common cancer, with incidence rates highest in Eastern Asia and sub-Saharan Africa. Risk factors for HCC development include hepatitis infection, alcoholic and non-alcoholic liver disease, cirrhosis, and exposure to aflatoxins (3).

Mutations within the genome drive the progression of both pancreatic and liver cancer. While some mutations are very commonly observed across multiple cancer patients, others are less frequent, representing heterogeneity within the mutational landscape. Both pancreatic and liver cancers show a high amount of somatic mutations, around 50 per tumor (4, 5). The presence and/or absence of specific mutations can dictate cancer therapy, and hence detection of the mutational profile of a given patient is a necessary step in effective treatment (6).

Tumor heterogeneity, an effect of genome instability, reduces the efficacy of targeted agents in personalized therapy, a therapeutic approach in which treatments are chosen based on the molecular basis of the disease in the individual (7). While markers for both PDAC and HCC exist, these markers have limitations which affect their clinical use. In PDAC, the most commonly used biomarker is elevated levels of carbohydrate antigen 19-9 (CA19-9), though this approach shows low positive predictive value for asymptomatic patients (<0.9%) despite high sensitivity (100%) and specificity (98.5%) (8). The most widely used HCC biomarker is serum alpha-fetoprotein (AFP), though its clinical use is limited by its lack of sensitivity (39–65%) and specificity (76–94%) (9, 10).

**Figure 1:**
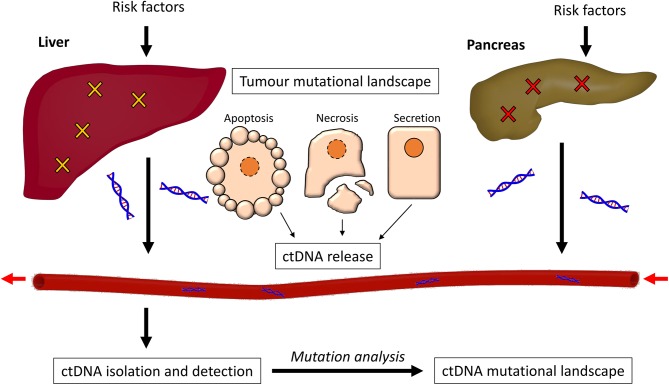
The process of ctDNA generation. Risk factors such as alcohol abuse and exposure to carcinogens promote somatic mutations in both the liver and the pancreas, causing cancer. The cancer cells can release ctDNA in three ways; through apoptosis, necrosis, or secretion. This ctDNA enters the bloodstream and can be isolated through purification methods. The mutational profile of the ctDNA is then detected and analyzed.

In this review, we discuss the mutational landscapes of both pancreatic and liver cancer, and how well they are represented by analysis of ctDNA. We begin by discussing the pathology of PDAC and HCC, and the signaling pathways on which these mutations converge. We then look at what is known about ctDNA and its release, and then discuss the methods used for isolation and analysis of ctDNA. We finally consider the many studies which have detected ctDNA in the analysis of PDAC or HCC, and the extent to which these studies can accurately identify mutations within the disease state (Figure 1).

## Pancreatic and Liver Cancer Pathology

PDAC and HCC development are both driven by somatic mutations, meaning they occur within an individual cell after conception and were not present in the previous generation. Driver mutations, which directly promote tumor growth, vary between different cancers, but tend to occur early on in disease development (11).

PDAC occurs in around 90% of pancreatic cancer cases, developing from normal acini, through precursor lesions, to ductal carcinoma. Mutations within pancreatic epithelial cells drive acinar-ductal reprogramming, and then the development of various stages of pancreatic intraepithelial neoplasia (PanIN) and then full PDAC. This development also involves the appearance of environmental characteristics such as desmoplasia, hypoxia, solid and fluid pressure, and autophagy (12).

**Figure 2:**
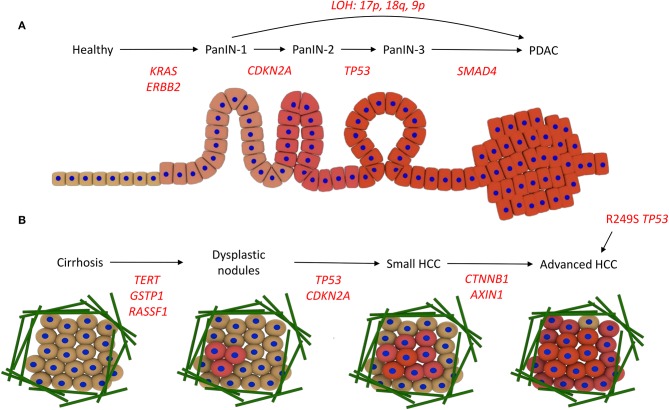
Driver mutations in PDAC and HCC development. **(A)** Development of PDAC. The acquisition of *KRAS* and *ERBB2* activating mutations drives progression to PanIN-1, *CDKN2A* promoter methylation and inactivity leads to PanIN-2, *TP53* inactivity promotes progression to PanIN-3, and then mutations in *SMAD4* lead to PDAC. Alternatively, loss of heterozygosity (LOH) promotes PanIN-1 to progress directly to PDAC. **(B)** Development of HCC. Various risk factors promote cirrhosis, involving the deposition of a large amount of ECM fibers (green). Mutations in the promoters for *TERT, GSTP1*, and *RASSF1A* drive the acquisition of dysplastic nodules, then mutations in *TP53* and the *CDKN2A* promoter promote small HCC development. Wnt signaling mutations, i.e., in *CTNNB1* and *AXIN1*, lead to advanced HCC. Alternatively, aflatoxin exposure can promote direct progression to HCC via the R249S mutation in *TP53*.

The development of PDAC, and its connection with the underlying genetic alterations of driver genes, is proposed to follow the multi-hit model. The first hit involves a mutation in the *KRAS* gene and overexpression of the receptor tyrosine kinase ErbB2 (*ERBB2* gene). Surviving cells are then altered by the second hit, in which the tumor suppressor gene *CDKN2A* becomes mutated through promoter methylation, leading to PanIN-1. Thirdly, tumor suppressor genes such as *TP53* and *SMAD4* become inactivated through mutation, leading to PanIN-2/3 and then PDAC (13–15). Loss of heterozygosity (LOH), where deletion of one of the copies of the gene occurs and therefore sensitizes the remaining copy to oncogenic mutations, is also linked to progression of different PanIN stages directly to PDAC. e.g., loss of heterozygosity at 17p, 18q, and 9p promotes PanIN-1 progression to PDAC (16) (Figure 2A).

The pathogenesis from healthy liver to HCC, the most common form of liver cancer, can be instigated in multiple ways. Chronic hepatitis B or C virus (HBV/HCV) infection, a diet rich in aflatoxins, or metabolic diseases can promote chronic hepatitis, which progresses to cirrhosis, a state often reached with high alcohol intake. This progression is associated with genetic instability. Cirrhosis precedes HCC in around 90% of patients and contains areas of abnormal hepatocytes known as dysplastic foci (<1 mm). These foci then develop into dysplastic nodules (>1 mm), and further develop into HCC. This progression from cirrhosis to HCC involves the accumulation of genetic and epigenetic alterations. Overexpression of *TERT*, and inactivation of *GSTP1* and *RASSF1A*, leads to the formation of dysplastic nodules following cirrhosis, and then inactivation of *TP53* and *CDKN2A* through mutation promotes HCC development. Wnt signaling pathway mutations (*CTNNB1* and *AXIN1*) occur at a later stage to promote advanced HCC (Figure 2B) (17, 18). Aflatoxin exposure can also promote progression to HCC without cirrhosis (19).

The downstream effects of the mutations which drive the progression of PDAC and HCC generally converge onto the pathways surrounding cell cycle regulation and oncogenic signaling (20). Progression through the cell cycle relies on a set of proteins which regulate various checkpoints, where activation must occur or the cell cycle is arrested. The different phases of the cell cycle are controlled by cyclins and the cyclin-dependent kinases (CDKs) they activate. These proteins oscillate in concentration during the phases of the cell cycle (21). Many effectors regulate the activity of these proteins, only allowing progression under certain cellular circumstances. Oncogenic signaling pathways in the cell promote survival and proliferation in response to external cues, such as growth factors or cytokines. Mutations in the effector proteins within these pathways, and those which regulate these effector proteins, are also common and lead to uncontrolled cell division.

## ctDNA Release

DNA can be released from multiple cells in different forms. As a highly charged molecule, DNA easily forms complexes with other molecules, and these structures protect the DNA from nuclease action and recognition by the immune system (22). Alternatively, DNA can attach to the external side of the cell membrane. Circulating DNA in healthy patients is generally double stranded and between 500 and 21,000 base pairs in length, whereas ctDNA is much smaller. Furthermore, the double stranded DNA derived from tumors has been shown to be less stable that that from non-tumor cells (23).

DNA is released from cells in processes associated with both health and disease. Living cells can secrete newly synthesized DNA as part of a protein complex, with many proteins implicated in this interaction including Argonaute2 and high density lipoprotein (24). It has been suggested that DNA within cells is regularly replaced to maintain a threshold level of DNA repair activity for maintenance of genome integrity (25). This controlled secretion may be important for ctDNA, as it has been observed that cell free DNA from breast cancer cells is released primarily through active secretion *in vitro* (26).

In contrast, apoptosis leads to the shedding of DNA as cell integrity is lost. Apoptosis involves a stepwise degradation of chromosomes into singular nucleosomal units, around which 146 base pairs of DNA are wrapped. DNA is then packed into apoptotic bodies to be shed, which are rapidly cleared by phagocytes. However, the pathology of cancer often inhibits the clearing ability of phagocytes (25). In necrosis, DNA degradation is much more random, and as such, releases DNA fragments of different sizes, up to 10,000 base pairs, which can be found in blood. Necrosis does not contribute to cell free DNA in healthy patients but does occur in tumor cells (27, 28).

Since there is increased DNA fragmentation in apoptosis compared to necrosis, apoptotic cell free DNA tends to be shorter, around the size of a nucleosomal unit. The size of ctDNA varies, with many fragments around 145–160 base pairs, suggesting apoptosis as an important mechanism. However, many fragments are smaller than 145 base pairs, indicating further degradation in the blood stream (29). Cell free DNA half-life is limited, as the spleen, liver, and kidney promote clearance, with an average half-life for cell-free fetal DNA of 16 min (30).

Concentrations of ctDNA increase with tumor stage and burden, but the overall proportion of cell free DNA that is tumor-derived can also be affected by the release of DNA from non-tumor cells, i.e., following lysis of white blood cells (31). Fragmentation of ctDNA is further increased as the tumor mass increases (32). Tumor location, size, and vascularity all affect the rate at which ctDNA is shed, though a lot of uncertainty still exists on how and why these effects occur, and this can influence the power of liquid biopsy tests (33).

Circulating DNA has a role in intercellular messaging independent of disease. One example of this is the co-operation between B and T lymphocytes in mediating the humoral immune reaction, in which T cell released DNA is suggested to provide the genetic information needed for B cells to synthesize the correct antibody (29). In contrast, genometastasis, the transfer of mutated DNA from one cell to another, is an oncogenic process that involves circulating DNA. For example, ctDNA from colon cancer has been shown to promote the oncogenic transformation of murine embryonic fibroblasts (34).

Other tumor biomarkers are also of interest in the field of liquid biopsy, and also contain genetic information which may shed light on the tumor mutational landscape. Circulating tumor cells (CTCs) have been suggested as a source of ctDNA, but these are unlikely to contribute much as CTCs are rare within the blood, and ctDNA is often present in the absence of CTCs (35). Exosomes, nanovesicles secreted by cells, contain DNA, but are also not suggested to contribute to ctDNA abundance (36).

## ctDNA Isolation and Detection

It is important to understand the variety of analysis methods for ctDNA, as appreciating their particular benefits and disadvantages allows a critical approach to the current set of studies and will improve the choices made in future ctDNA research.

ctDNA analysis is able to detect point mutations and copy number variation, but unable to detect larger scale mutations such as chromosomal aberrations (1). The concept of genomic variation as a guiding marker for therapy selection has been previously demonstrated, e.g., the variation in the gene *SLC15A2* as a marker for responsiveness to sorafenib in HCC (37). In addition, ctDNA analysis, for detection of the T790M mutation in the epidermal growth factor receptor (EGFR), has been recently used to guide therapy selection in non-small lung cancer (38).

ctDNA can be isolated from multiple body fluids, but most is most commonly extracted from blood plasma or serum. Pancreatic juice, bile, saliva, urine, and pleural effusion can also be used as a source of ctDNA (36). In one isolation method, guanidinium-thiocyanate-phenol-chloroform is used to separate RNA from DNA under acidic conditions, where DNA remains in the organic phase whereas RNA remains in the aqueous phase (39). Other common DNA isolation methods use kits that involve silica-based columns, polymer-mediated enrichment, or magnetic beads (40). Other pre-analytical variables that should be considered include the choice of body fluid, collection and processing materials, storage conditions and thawing temperatures (41).

### Specific Mutation Detection

Amplification of ctDNA requires faithful duplication of the DNA sequence. Quantitative PCR or real-time PCR (qPCR) is used to exponentially amplify a segment of DNA and concurrently quantify levels of DNA. Primers are designed to flank the sequence to be amplified, e.g., a specific exon of a gene, are therefore independent of the presence of a mutation.

**Figure 3:**
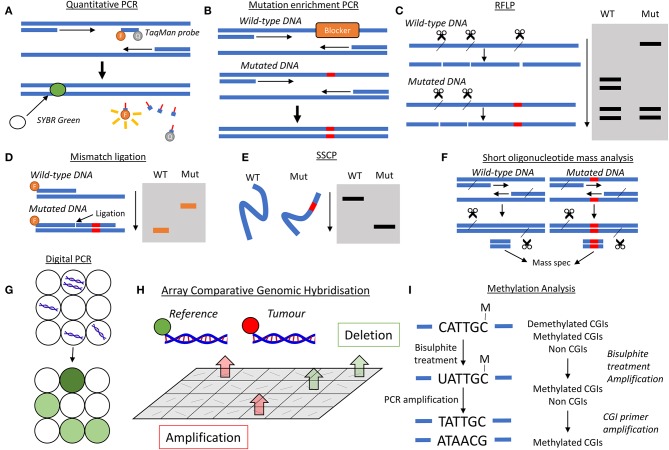
Analysis methods to ctDNA analysis. **(A)** Quantitative PCR, either with a TaqMan probe or a double stranded DNA probe such as SYBR green. TaqMan probes bind to regions of interest, which could be a specific mutation, and nuclease activity during amplification separates the fluorophore and quencher, leading to fluorescence. SYBR green is unspecific for sequence, but binds to double stranded DNA and becomes fluorescence, which can be detected. **(B)** Mutation enrichment PCR. A blocking molecule binds to the wild-type sequence preventing its amplification. Therefore, only mutated DNA is amplified. **(C)** Restriction fragment length polymorphism (RFLP). If mutations alter the short sequences recognized by nucleases, then the fragment profile differs between wild-type and mutated DNA. When run on an electrophoresis gel where fragments are separated by length, the band profile will differ between wild-type and mutated DNA. **(D)** Mismatch ligation. Two probes are used, one attached to a fluorophore and another with a sequence to detect the mutation. By adding a ligase enzyme, longer fragments are generated for mutated DNA, which run differently on an electrophoresis gel. **(E)** Single strand conformation polymorphism. Wild-type (WT) and mutated DNA will form slightly different conformations are single strands, leading to a different movement on an electrophoresis gel. **(F)** Short oligonucleotide mass analysis. A very short fragment, around 7 base pairs, from the gene of interest is generated and subjected to mass spectrometry. **(G)** Digital PCR. The DNA solution is separated into many discrete volumes, containing none or some of the DNA. This DNA is amplified and the proportion of DNA positive volumes is used to quantify ctDNA levels. **(H)** Array comparative genomic hybridization. Reference and tumor DNA are labeled differently and allowed to bind to an array of DNA targets from a library. If deletion has occurred in the tumor, more reference DNA will attach to a particular sequence. If amplification has occurred in the tumor, more tumor DNA will attach to a particular sequence. These changes manifest in different intensities of each label. **(I)** Methylation analysis. Left: bisulfite treatment leads to unmethylated cytosine residues becoming uridine residues. When amplified, these Us pair with adenine residues, so any cytosines present following amplification are those that were methylated. Right: methylated CpG tandem amplification sequencing (MCTA-Seq) isolates methylated CpG islands (CGIs) for analysis of global genome methylation.

TaqMan PCR and SYBR green analysis allow for real-time quantitative analysis of PCR amplification (Figure 3A). In TaqMan PCR, a probe is designed to bind a specific sequence of interest and contains both a fluorophore and a quencher probe located near each other, hence no fluorescence is observed. If the sequence of interest is present, the probe binds to that sequence, and then PCR amplification leads to degradation of the probe through 5′ to 3′ exonuclease activity. This separates the fluorophore and quencher, leading to fluorescence. SYBR green analysis is not specific to any DNA sequence but becomes fluorescent upon binding to the minor groove of double stranded DNA, where more binding sites are created with PCR amplification. The SYBR green assay is low cost and easy to use, though can suffer from a lack of specificity. The sensitivity of TaqMan probes are similar to SYBR green, but do show an increased specificity (42).

Amplification of only mutant alleles can be achieved through mutation enrichment PCR (Figure 3B). In this method, a blocking segment of DNA is used that only binds to the wild-type version of the gene, and its presence blocks the progression of DNA polymerase. Where a mutation has occurred, this blocking segment does not bind and therefore DNA polymerase is able to amplify this DNA region (43). A further development is PNA-PCR clamping, in which peptide nucleic acids (PNA) are used to bind more strongly to specific sequences of DNA to block PCR amplification. Locked nucleic acids and morpholinos can also be used for this purpose (44). This specific amplification is often combined with a non-specific quantification method e.g., SYBR green.

Restriction fragment length polymorphism (RFLP) can also be used to detect mutations (Figure 3C). The premise behind the analysis technique is that alterations of bases in DNA change the interaction of various nucleases with the DNA. If a nuclease cannot bind, then cleavage at that site does not occur, leading to a difference in the fragment profile following nuclease treatment. Therefore, if a mutation occurs in a nuclease binding site, then the wild-type DNA will be cleaved whereas the mutated DNA will not, leading to polymorphism, i.e., a difference in the length of the variety of restriction fragments (45). Many studies have used RFLP in DNA analysis, including the detection of mutations in the gene *TP53* in HCC (46).

The mismatch ligation assay involves the use of DNA probes that target mutated sequences, as well as labeled probes (Figure 3D). Both probes are allowed to attach, where the mutant probe only attaches in the presence of a mutation. A DNA ligase enzyme is added to ligate the two probes into one, which is then removed. The probes are then run on a gel, where movement is dependent on DNA size, and detected. If a mutation is present in the analyzed DNA, then the ligation produces a longer labeled fragment, and therefore the longer probe moves differently within the gel. This method has been used to analyse common mutations in pancreatic cancer (47).

Mutations in pancreatic cancer have also been analyzed through single strand conformation polymorphism (SSCP) (Figure 3E) from DNA from pancreatic juice (48). In SSCP analysis, the gene of interest is amplified using PCR, denatured into single strands and then run on an electrophoresis gel. The slight differences in sequence due to mutations affect the conformation of the single strands, altering their movement within the gel (49).

In short oligonucleotide mass analysis (Figure 3F), a short region of the genome (as small as 7 base pairs) is amplified by PCR, with the primers engineered to contain an endonuclease site. Following amplification, digestion of the PCR product leaves only the short genomic region, which is subsequently analyzed by electrospray ionization mass spectrometry to determine its sequence (50). This method has been used to assess a specific *TP53* mutation in HCC (51).

Digital PCR is a recent development which increases sensitivity. This process involves separating DNA templates into discrete volumes, such that some contain no DNA template and some contain at least one DNA template (Figure 3G). PCR amplification is then performed, so that the volumes with a relevant DNA template will be amplified whereas those without will not be amplified. The number of DNA positive volumes following PCR amplification, often determined with the TaqMan assay, is then used to calculate the DNA concentration. In droplet digital PCR (ddPCR), the discrete volumes are oil droplets within a water-based solution (52). Heterogeneity in the mutation profiles of ctDNA of HCC patients has been demonstrated through this assay (53).

Since, the copy number of genes can be altered by amplification or deletion mutations, methods for analyzing copy number such as Array Comparative Genomic Hybridization (aCGH) have been developed (Figure 3H). In this method, tumor DNA is labeled with one fluorophore and reference DNA from a healthy sample is labeled with another fluorophore. These DNA solutions are then mixed and added to an array of DNA targets. If deletion has occurred, then there will be more reference DNA attached to a specific DNA target, and if amplification has occurred, then there will be more tumor DNA attached to a specific DNA target (54). The copy number of various genes in the ctDNA of breast cancer has been analyzed this way (55).

### Methylation Detection

Since, many reported mutations in both pancreatic and liver cancer involve aberrant methylation of specific gene promoter regions, detection of these mutations within ctDNA must use specific techniques to maintain a marker of methylation during PCR amplification, most commonly sodium bisulfite treatment (Figure 3I). Methylation occurs primarily on the C5 position of cytosine bases within the cytosine-guanine dinucleotide (CpG). The product, 5-methylcytosine, is unaffected by treatment with sodium bisulfite, whereas unmethylated cytosine residues are converted into uracil. PCR amplification converts uracil bases into thymine bases, and therefore when the PCR product is sequenced, any cytosine residues present are those that were methylated in the original DNA (56). One interesting technique is known as “methylation on beads,” which combines DNA extraction, bisulfite conversion and PCR in one tube using silica superparamagnetic beads, and has been used to analyse the promoter region of the *CDKN2A* gene in lung cancer (57).

Global methylation is not suited for bisulfite analysis as large amounts of DNA would be needed to represent the whole genome. Shotgun massively parallel bisulfite sequencing has been developed, a sequencing platform with high throughput and has been used to assess global hypomethylation in HCC (58). Another analysis technique for methylation across the genome is methylated CpG tandem amplification and sequencing (MCTA-Seq) (Figure 3I). This method looks at the methylation state of the 7-mer CGCGCGG, also known as a CpG island, which is common in the genome. In the 1st step, following bisulfite treatment, unmethylated sequences are eliminated as they are amplified less than methylated sequences by a specific primer. Methylated sequences, but those that are not CpG islands, are then eliminated by a CpG island specific primer. The product, containing only methylated CpG islands, is then amplified for quantification purposes (59).

### Sequencing Analysis

For larger scale analyses, next-generation sequencing (NGS) is increasingly used. NGS involves the sequencing of millions of short fragments of DNA in parallel, and multiple platforms have been developed for this high throughput analysis technique (60). The detailed mechanisms of the wide variety of NGS platforms is outside the scope of this review but have been well-reviewed elsewhere (61, 62). The main difference between NGS analysis of DNA directly from cells and ctDNA analysis is the lack of a ctDNA fragmentation step in the preparation of a DNA library, as ctDNA is already present in small fragments. NGS methods can also be used for analysis of copy number in ctDNA (63).

One NGS platform that has been recently used for analysis of mutations in ctDNA from HCC patients is Guardant360. This platform uses a panel of oncogenes and tumor suppressor genes and analyses each ctDNA sample set for single nucleotide variants, copy number amplification and other fusion and insertion mutations. The assay reports the type of mutation, if present, for each gene for each patient (64).

Other NGS assays using gene panels, currently in development for other cancer types, may also show promise for either pancreatic or liver cancer in the future. For example, the Cobas® EGFR Mutation Test v2 is used for non-small-cell lung carcinoma since EGFR, a KRAS-activating receptor, is often mutated in this particular cancer (65). Another NGS platform, the PlasmaSELECT-R64 assay, evaluates a panel of 64 genes and has been directly compared with the Guardant360 assay on samples from patients with metastatic prostate cancer. In this study, the genomic alterations observed varied greatly depending on the assay used despite an overlap of genes tested, and these inconsistencies mean that the effectiveness of personalized medicine could vary depending on the NGS platform used (66).

## ctDNA Mutational Landscape and Detection

PDAC and HCC have characteristic mutational landscapes, where some genes are hotspots for driver mutations which facilitate disease progression. Though these two gastrointestinal diseases share many common genetic alterations, some are specific to each pathology. For example, both pathologies commonly show mutations in the genes *TP53* and *CDKN2A*. In addition to these shared alterations, PDAC frequently exhibits mutations in *KRAS*, as well as *ERBB2* and *SMAD4*, whereas HCC is often characterized with mutations in the *TERT* promoter, *CTNNB1* and *AXIN-1*.

Only a subset of the mutations present in the tumor mutational landscapes of PDAC and HCC have been detected by studies which have analyzed ctDNA in the body fluids of cancer patients. The metrics commonly used in ctDNA analysis are absolute values for ctDNA abundance (either specific to a target gene or overall ctDNA levels) or the percentage of patients in a cohort with a particular mutation. For diagnosis purposes, a mutation must be highly sensitive, in that its detection indicates the presence of the disease, but also highly specific, in that lack of its detection indicates the absence of the disease. Prognosis involves relating mutations or ctDNA abundance to clinical metrics such as overall survival or time to relapse.

Here we describe what is currently known about the mutational landscapes of PDAC and HCC, and how well recent studies have been able to represent this landscape through analysis of ctDNA.

### Cell Cycle

#### TP53

**Figure 4:**
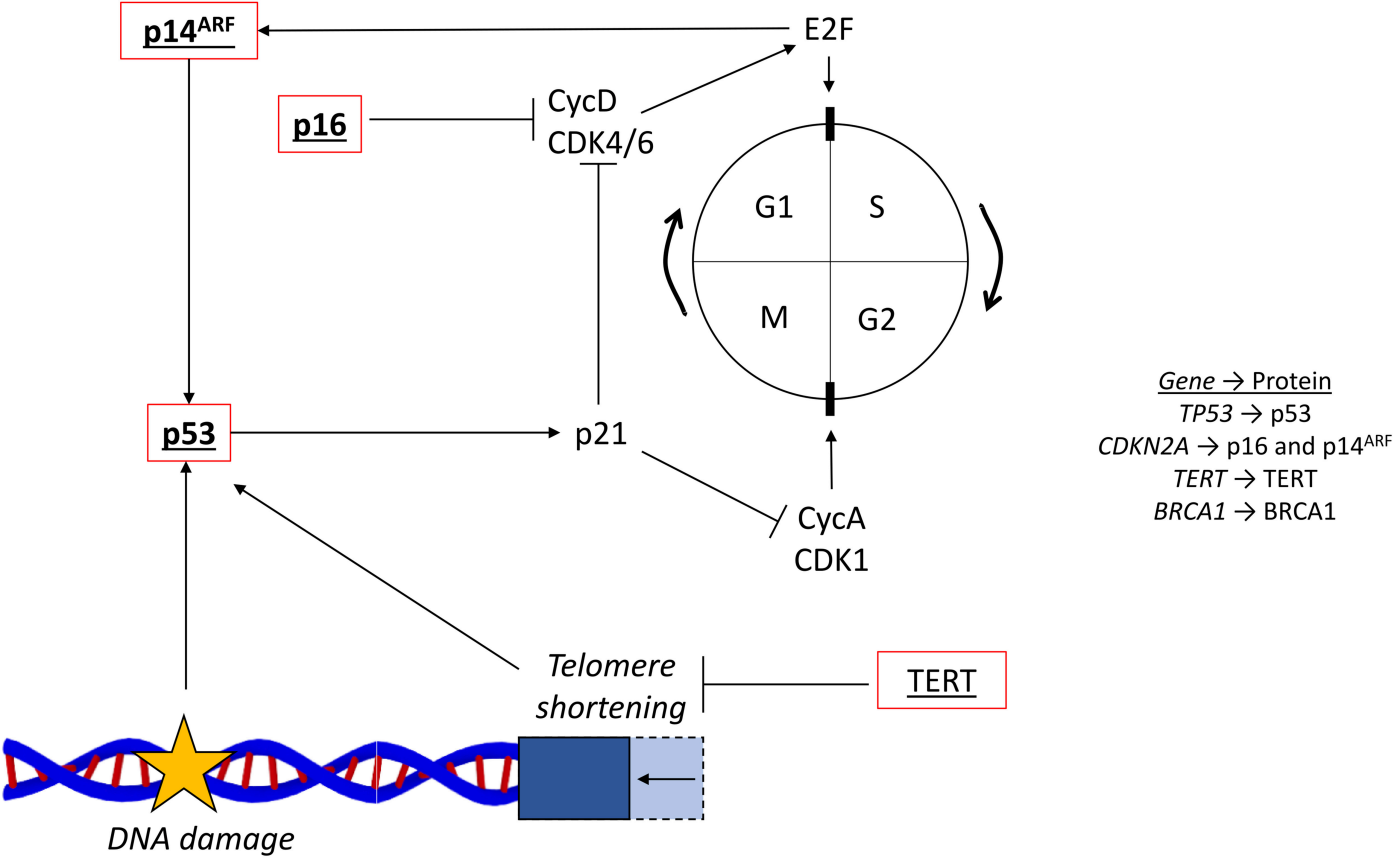
Cell cycle and common mutations. The cell cycle (…G1 → S → G2 → M → G1…) is regulated by cyclins and their associated cyclin-dependent kinases. The G1/S transition is controlled by CycD and CDK4/6, which activates E2F. The G2/M transition is controlled by CycA and CDK1. The tumor suppressor p53 responds to DNA damage and activates p21, which inhibits cell cycle progression via the cyclins. Telomere shortening, reversed by the activity of TERT, also activates p53. The two *CDKN2A* coded proteins, p16 and p14ARF, are also involved in the regulation surrounding E2F, CycD, and CDK4/6. Red outline, commonly downregulated in tumors; bold, commonly mutated in PDAC; underlined, commonly mutated in HCC; bold and underlined, commonly mutated in both PDAC and HCC; CycD, cyclin D; CDK4/6, cyclin dependent kinase 4/6; CycA, cyclin A; CDK1, cycling dependent kinase 1; TERT, telomerase reverse-transcriptase; NFE2L2, nuclear factor erythroid 2-related factor 2.

One role of each cell cycle checkpoint is to ensure that there is no DNA damage before cell cycle progression occurs. If arrest at these checkpoints is not properly controlled by the multitude of signaling proteins involved in their regulation, then cancer can develop (67). One key DNA damage response protein is p53, coded for by the gene *TP53* located on chromosome 17. p53, a commonly mutated tumor suppressor, is activated by DNA damage, leading to transcriptional upregulation of its target genes to halt the cell cycle. For example, by promoting the expression of p21, which inhibits multiple cyclins and their CDKS, p53 inhibits progression through both the G1/S and G2/M checkpoints (Figure 4) (68).

Mutations in *TP53* are present in HCC and exist in 35–50% of patients (69, 70). The most common missense mutation is R249S and is linked to exposure to the mycotoxin aflatoxin B_1_, which can promote both cirrhosis-dependent and independent progression to HCC (Figure 2B). Furthermore, the HBx protein, expressed from insertion of the hepatitis B virus into the genome in HCC, has been shown to inhibit the activity of the wild-type p53 protein (71). The ctDNA analysis of mutations in the *TP53* gene for HCC diagnosis has mostly analyzed populations with a high dietary exposure to aflatoxin B_1_. The R249S mutation has been detected at a higher level in ctDNA from HCC patients than in healthy controls in a variety of studies, suggesting its diagnostic potential, and has been also been shown to be associated with worse survival than wild-type *TP53* (72).

In a 2000 study in The Gambia, the R249S mutation was detected in 36% of HCC patients but in only 6% of healthy controls (73). In Nigeria, the same approach showed a smaller detection rate of 8% in HCC and 0% in healthy controls (74). Another African study, which analyzed data from a variety of tribal groups, detected this mutation in 18% of their cohort of 158 black southern Africans (75). In contrast, an analysis of patients in Egypt detected this *TP53* mutation in only 1.3% of HCC cases and 1.4% of healthy cases, though higher levels (17%) were detected in chronic liver disease cases (46).

As well as Africa, Asian regions have also been studied as places where aflatoxin β_1_-mediated mutation of *TP53* occurs. In the Qidong region of China, the R249S mutation was detected in HCC cases in 2003 at a sensitivity of 44% and specificity of 93% (76). A study in Thailand in 2005 found the mutation R249S in 26% of HCC cases but only 15% of healthy cases (51). These studies indicate that the detection of the R249S mutation shows promise but may only be highly specific for HCC in certain regions.

Quantitative analysis of the plasma concentration of R249S *TP53* has also been performed for HCC and healthy cases. A 2005 study from The Gambia determined that the median concentration of R249S *TP53* in HCC cases (2,800 copies/mL) was higher than that of cirrhotic or healthy cases (both 500 copies/mL). HCC diagnosis was significantly associated with >10,000 copies of R249S *TP53* per mL (77).

*TP53* is inactivated in 20–76% of pancreatic cancers, primarily through a mutation in one allele along with loss of the other allele. Many of these mutations occur in the DNA binding domain of p53 (78, 79). Mutations in *TP53* cannot initiate pancreatic cancer (13) and tend to appear in later stage PanINs (Figure 2A). Only a few studies have looked at ctDNA *TP53* mutations for the analysis of PDAC, despite mutations occurring abundantly in the tumor. One study in 2017 used NGS to identify a range of *TP53* mutations from ctDNA in pancreatic juice with high specificity. In 59% of PDAC patients, *TP53* showed some sort of mutation, whereas no control cases exhibited any alterations (80). The aflatoxin β_1_-mediated mutation of R249S has additionally been detected in ctDNA from pancreatic cancer patients in Iran in a 2013 study, with an 11% incidence in pancreatic cancer but only 3.5% of healthy cases (81). Furthermore, the *TP53* mutations, I251M and R175G, have been detected in the ctDNA of individual pancreatic cancer patients pre-surgery, and the mutations G293E, M340Cfs^*^5, S362Afs^*^8, and T211I have been detected in individual patients who developed metastasis after resection of the primary tumor (82).

#### CDKN2A

Another tumor suppressor gene that is commonly mutated, *CDKN2A*, encodes two other cell cycle regulatory proteins, p16 and p14^ARF^. *CDKN2A* is situated on the short arm of chromosome 9, with p16 and p14^ARF^ generated from different reading frames. Inactivation of the *CDKN2A* promoter by hypermethylation is a common occurrence in both HCC and PDAC (78). p16 has a key role in regulation of the G1/S checkpoint in the cell cycle, and p14^ARF^ is involved in activating p53 (Figure 4) (83).

From an analysis of 71 HCCs, it has been shown that 66% of HCC cases exhibit inactivation of p16 and 15% exhibit inactivation of p14^ARF^ (69). Most commonly, the *CDKN2A* promoter is methylated, leading to inactivation, and this hypermethylation has been observed on average in 58% of HCC cases (84). While promoter methylation is the dominant form of mutation, missense and nonsense mutation of *CDKN2A* have also been seen in liver cancers, including H75Y (85) and R58^*^ (86). Additionally, 7% of HCC cases show homozygous deletion of the *CDKN2A* gene (69).

*CDKN2A* alterations have been detected in the ctDNA of HCC patients in many studies. A 2003 study detected methylation of the *CDKN2A* promoter in the ctDNA of 47% of HCC patients where promoter methylation had been observed in the tumor (87). Other studies by the same group detected *CDKN2A* promoter methylation in around 80% of HCC patients where methylation was present in the tumor. ctDNA methylation was not detected in any patients where none was present in the tumor in both studies (87–89). Furthermore, promoter methylation was observed in the plasma of liver cancer patients pre-surgery at a rate of 31% and the median amount of methylation of the *p16* genes analyzed was 12-fold lower following surgery (87). NGS methods on ctDNA from HCC patients have also detected the presence of the *CDKN2A* mutant R80^*^ (64).

In pancreatic cancers, *CDKN2A* is inactivated in ~40% of cases by deletion of both alleles (78), with loss associated with worse survival probability (90). Inactivation occurs in a further 40% by deletion of one allele and a mutation within the remaining allele (91). Furthermore, 15% of pancreatic cancers show hypermethylation of the promoter sequence for *CDKN2A* (78, 92). ctDNA analysis of *CDKN2A* for pancreatic cancer is limited, though one study identified mutations from DNA in pancreatic juice at an incidence of 6% in PDAC and 0% in control cases (80).

#### TERT

Telomeres are nucleoprotein structures, located at the tip of each chromosome, which protect chromosome ends from fusion, recombination and degradation. Telomeres shorten with every cell cycle, ~50–150 base pairs per cycle, and when they reach a critically short length, promote cell cycle arrest by activating p53 (Figure 4) (93). The gene *TERT* encodes telomerase reverse-transcriptase (TERT), which extends telomeres. Increased activation of TERT therefore promotes the lengthening of telomeres and increases cell growth (94). Upregulation of *TERT* is common in HCC, most commonly through activating mutations within its promoter region (95).

The *TERT* promoter is the most frequently mutated site in HCC, with ~60% of cases exhibiting alterations, most frequently at the positions 124 and 146 base pairs upstream of the ATG start site. Both sites involve a mutation of a guanine to an adenine, and additionally, position 124 has been shown to mutate a guanine to a thymine (95). This creates a binding site for transcription factors of the ETS family which promote TERT expression (96). A further 10–15% of TERT reactivation occurs through insertion of hepatitis B virus into its promoter, and 5% is due to *TERT* amplification (5).

Despite *TERT* promoter mutations driving the initial progression of HCC (Figure 2B), and being highly abundant in liver cancers, their detection within the ctDNA landscape has been limited. The specific mutations in the promoter region for TERT that enhance ETS binding, as seen in HCC biopsies, have yet to be detected through ctDNA analysis. Some studies have used *TERT* DNA as an amplification locus for the quantification of overall levels of ctDNA instead of analyzing mutations in the promoter. One study used real-time PCR to show that the abundance of *TERT* DNA in HCC patients was higher than that of HBV patients and healthy controls, though *TERT* abundance was not associated with tumor size or stage (97). Another study observed that *TERT* levels were significantly associated with reduced overall survival, having analyzed concentrations of *TERT* DNA in patients with HCC, cirrhosis and chronic hepatitis (98).

A study of multiple cancers has revealed that PDAC and pancreatic acinar carcinoma do not show *TERT* mutations (99).

### Oncogenic Signaling

#### KRAS

**Figure 5:**
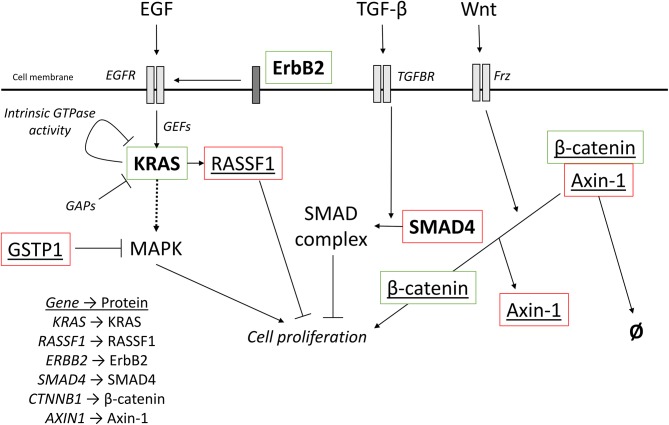
Oncogenic signaling pathways with driver mutations for PDAC and HCC. External signaling molecules, such as EGF, TGF-β, and Wnt, promote intracellular signaling through their respective receptors, EGFR, TGFBR, and Frz. EGFR activation, facilitated by ErbB2, leads to activation of KRAS. KRAS is inactivated by either intrinsic GTPase activity or GAPs. Cell proliferation can be promoted by the MAPK cascade is activated by KRAS but can also be inhibited by the redox regulator GSTP1. KRAS can also activate RASSF1A, which inhibits cell proliferation. TGF-β signaling promotes the incorporation of SMAD4 into a heterotrimeric complex with inhibits cell proliferation. Wnt signaling activated the Frz receptor, which promotes the decoupling of Axin-1 from β-catenin. In the complex, β-catenin is targeted for degradation, but when not complexed, β-catenin promotes cell proliferation. Red outline, commonly downregulated in tumors; green outline, commonly upregulated in tumors; bold, commonly mutated in PDAC; underlined, commonly mutated in HCC; bold and underlined, commonly mutated in both PDAC and HCC. EGF, epidermal growth factor; EGFR, EGF receptor; TGF-β, transforming growth factor β; TGFBR, TGF-β receptor; Frz, Frizzled; GEFs, guanine nucleotide exchange factors; GAPs, GTPase activating proteins.

Located on chromosome 12, the *KRAS* gene codes for the 21 kDa GTPase KRAS and is mutated in over 90% of pancreatic cancers (78). If constitutively activated by mutation, KRAS promotes oncogenic signaling through multiple signaling pathways. In its wild-type form, KRAS is activated by cell surface receptors such as the EGFR, leading to activation of the MAP kinase cascade to promote cell proliferation, metabolism and transcription of target genes. KRAS is activated by guanine nucleotide exchange factors (GEFs) which exchange bound GDP for GTP, and is then deactivated either by GTPase activating proteins (GAPs) or through its intrinsic GTPase activity (Figure 5) (100).

Ninety eight percentage of *KRAS* mutations affect the glycine residue at position 12, with missense mutations swapping glycine for aspartate, valine, or arginine. This alteration blocks the intrinsic GTPase activity of KRAS and makes the molecule insensitive to GAPs, leading to constitutive activation (100, 101). A few mutations (overall <2%) also occur at positions 13, 61, 117, and 146 (79). Mutations occur in around 30% of early neoplasms, increasing to around 95% of advanced carcinomas (102, 103). Mouse models use the mutation *KRAS*^*G*12*D*^ to initiate PDAC development and have been used to demonstrate that this mutation in one of the earliest events in PanIN initiation in humans (Figure 2A) (101).

As the most commonly mutated gene in pancreatic cancer, detection of mutated *KRAS* in ctDNA is a highly studied area. *KRAS* mutations have been detected in plasma or serum DNA at a range of incidence rates (from 33 to 94%) (47, 80, 104–107).

A 94% sensitivity was seen in a 2017 study on 189 patients with unresectable PDAC using mutation enrichment PCR, following by NGS, to identify G12 mutations. The most common mutation amongst these was G12D (41%). This study also showed that concentrations of *KRAS* ctDNA were increased in stage IV PDAC patients compared with stage III, and these high values were significantly associated with shorter overall survival (107).

The lowest sensitivity observed, 33%, was seen from plasma DNA samples from PDAC patients in China using PCR. Though a low sensitivity was observed, the presence of mutations significantly reflected clinical parameters, including tumor stage and the presence of liver metastasis. The survival time for patients was also significantly negatively associated with the presence of *KRAS* mutations (105). A similarly low sensitivity of 35% was observed using a sensitive mutation specific mismatch ligation assay on plasma DNA from pancreatic cancer patients (47).

Further delineation of the presence of *KRAS* mutations in different stages of pancreatic cancer has been performed. Mutations have been shown to be more abundant in patients with metastatic disease (90%) than local disease (43%) (106), and the use of *KRAS* mutations to differentiate between pancreatic cancer and chronic pancreatitis has been demonstrated with ctDNA analysis at a sensitivity of 47% and specificity of 87% (104).

*KRAS* mutations, as detected with ctDNA, have also been associated with poor survival in pancreatic cancer. Using ddPCR and amplification of mutant DNA with TaqMan probes for various *KRAS* G12 mutations, ctDNA abundance in PDAC patients has been significantly associated with reduced overall survival (108). Another study used a PNA clamp specific for the wild-type *KRAS* sequence to perform mutation enrichment PCR for patients about to undergo a chemotherapy regime for PDAC. Pre-therapy mutant *KRAS* ctDNA abundance was significantly associated with reduced progression-free and overall survival (109).

#### RASSF1A

*KRAS* mutations have been suggested to not contribute to the pathogenesis of HCC (110). However, RASSF1, a downstream target of Ras family members (Figure 5) and an often mutated tumor suppressor protein, is associated with liver cancer (111). Hypermethylation of the *RASSF1* promoter, leading to downregulation of expression, occurs in HCC at a rate of 93% (112). The RASSF1 protein is also a negative regulator of the Hippo pathway, which promotes cell growth (113). Methylation of the *RASSF1* promoter has been seen in pancreatic cancer and is present in 35% of tumors. However, the phenotypic result of this is a variation in the expression of different isoforms of RASSF1 and is not associated with prognosis (114).

*RASSF1A* has garnered attention for ctDNA analysis and *RASSF1A* promoter methylation has been observed in the analysis of serum DNA in HCC. As part of a longitudinal study, hypermethylation was observed as present up to 9 years before the clinical diagnosis of HCC. Out of the HCC cases, 70% showed *RASSF1* promoter hypermethylation in ctDNA (115). Hypermethylation of the *RASSF1A* promoter has been associated with HCC size of >4 cm (112). Additionally, poorer disease-free survival has been associated with hypermethylation, and this increase was observed in ctDNA longitudinally for patients who carry the hepatitis B virus from enrolment to HCC diagnosis (116).

#### ERBB2

Various cell membrane receptors promote activation of Ras family members, and one of these receptors which is commonly mutated is the erythroblastic oncogene B2, known as ErbB2 (Figure 5). The gene for ErbB2 (*ERBB2)* is located on chromosome 17 and is sometimes referred to as *HER2* (117). ErbB2 expression has been shown to be very low in healthy pancreatic ducts but high incidence has been observed in various ductal malignancies (118).

The *ERBB2* gene is frequently overexpressed in PDAC, and this is associated with a worse prognosis. ErbB2 additionally modulates the resistance of pancreatic cancer cells to the chemotherapeutic gemcitabine (119). Protein overexpression of ErbB2 has been seen at a variety of incidence rates, ranging from 7 (120) to 61% (121). Amplification of the *ERBB2* gene has also been observed at incidence rates from 2 (120) to 24% (122). Furthermore, missense mutations have also been observed in pancreatic cancer, including R103Q, V8421I, and E717D (123).

Despite mutations in the gene *ERBB2* being highly associated with the early stages of PDAC, its mutational status within the ctDNA landscape is less well-considered. Using NGS to detect mutations, and then ddPCR for further analysis, mutations in *ERBB2* (either in exon 17 or 27) have been detected in the ctDNA of 20% of pancreatic cancer patients. Exon 17 mutations were additionally associated with significantly reduced overall survival (124). Amplification of the *ERBB2* gene in pancreatic cancer has also been observed by ctDNA analysis (125).

In HCC, *ERBB2* is rarely altered. One missense mutation (H878Y) has been observed in liver cancer at an incidence of 11% (126). An ErbB effector, ERRFI1, has been reported as being mutated in 5% of HCC cases (70, 127). A literature review which reviewed mutations in a multitude of cancer types did not report any studies where *ERBB2* was mutated in liver cancer ctDNA (128). However, genomic alterations in *ERBB2* are found at a rate of 25% in extrahepatic cholangiocarcinoma, but not in intrahepatic cholangiocarcinoma (129). Cholangiocarcinoma (CC) is a common type of liver cancer which begins in the bile ducts which connect the liver to the gallbladder, and cases are classified as intra- or extrahepatic depending on which part of the biliary system they arise in Massarweh and El-Serag (130). Future ctDNA testing for *ERBB2* may therefore be useful for some CC cases, as well as those rarer cases where patients have the mixed malignancy where both HCC and CC are present.

#### SMAD4

Another oncogenic signaling pathway is the TGF-β pathway, and involves the effector protein SMAD4, which is often inactivated through mutation in PDAC. With its gene located on the long arm of chromosome 18, SMAD4 promotes inhibition of epithelial cell growth (131). Extracellular transforming growth factor beta (TGF-β) promotes the formation of SMAD complexes, with SMAD4 a subunit of a heterotrimer which promotes expression of tumor suppressor genes (Figure 5) (132). *SMAD4* is inactivated in 35% of pancreatic cancers by homozygous deletion (78), where loss is a negative prognostic indicator and associated with poor survival (133).

As a low abundance mutation, detection of *SMAD4* mutations has generally proceeded through NGS approaches. Using digital NGS to efficiently sequence low-abundance mutations, *SMAD4* mutations, either frameshift or missense, have been identified from DNA in pancreatic juice at an incidence of 15% in PDAC and 0% in control cases (80). Using targeted resequencing to focus on specific genes for amplification and analysis, NGS has also been used to demonstrate that *SMAD4* mutations were present in the ctDNA of only 5% of PDAC patients (134).

#### Wnt Signaling (CTNNB1 and AXIN1)

The Wnt signaling pathway also transduces extracellular signals which affect cell development and is closely associated with cancer. Canonical Wnt signaling involves an AXIN-containing protein complex that promotes the degradation of the signaling effector β-catenin, coded for by the gene *CTNNB1*. Upon activation of the pathway by extracellular Wnt ligand, this complex is disrupted and β-catenin translocates to the nucleus to regulate gene expression (Figure 5).

Activating mutations of *CTNNB1* occur at a rate of 11–37% in HCC (5). Large in-frame deletions in exon 3, and missense mutations between residues 32 and 37 lead to high levels of β-catenin activation, as they prevent the binding of β-Trcp which would promote ubiquitination and degradation. Other mutations, such as those involving Ser45, lead to weak activation of β-catenin as they block a phosphorylation site that promotes degradation. Ser45 mutations only lead to development of benign tumors, but selective duplication of this mutated allele and production of double the dose of mutated β-catenin is suggested to promote progression to a malignant tumor (135).

Mutations in *CTNNB1*, with the nucleotide changes A121G and T133C, have been detected in 13% of the ctDNA of HCC patients using ddPCR with primers specific for certain mutations (53). With NGS, *CTNNB1* mutations leading to the amino acid changes of S29T, S33C, H36P, and G34V were detected in 29% of HCC patients (64). A previous study published by the same group that year analyzed ctDNA in a further 26 patients with HCC using NGS and demonstrated that 31% of HCC patients showed *CTNNB1* mutations. These were missense mutations leading to the amino acid changes D32N, S45P, S45F, S37F, T41A, as well as S33C, H36P, and G34V which were observed in the group's later study (136).

Axin-1 is a protein involved in the protein complex that regulates β-catenin and is coded for by the *AXIN1* gene. In a study involving 100 HCC cases, *AXIN1* mutations were observed at a rate of 6%, including nonsense and frameshift mutations. These mutations are predicted to truncate Axin-1 to remove the β-catenin binding site, and therefore Axin-1 is no longer able to facilitate β-catenin degradation (137). However, *AXIN1* mutations have not been detected in ctDNA from HCC patients (136).

#### GSTP1

One protein that can regulate kinases within oncogenic signaling pathways is glutathione S-transferase π (GSTP1). The main role of GSTP1 is to detoxify the cytoplasm by conjugating with xenobiotics and maintaining redox homeostasis. If GSTP1 expression is reduced, carcinogen detoxification is diminished and therefore genome instability is promoted. GTSP1 has the additional role of negatively regulating kinases that act as effectors which promote cell proliferation (Figure 5) e.g., MAPK (138) and c-Jun (139).

Downregulation of expression from *GSTP1* occurs in HCC through methylation of its promoter region in around 53% of HCC cases (140), with methylation of certain regions occurring more often than others, and more specifically in HCC. For example, methylation in one promoter region has been shown in 77% of HCC cases and no healthy cases, and methylation in another region of the promoter has been shown in 80% of HCC cases, but also in 100% of healthy cases (141). High levels of methylation of the specific region (5' of−48) of the *GSTP1* promoter are more abundant in HCC (37%), compared to other liver conditions including hepatitis, cirrhosis, as well as healthy control (all 0%), and only 15% of HCC cases show no methylation (141). The *GSTP1* promoter region has also been shown to be methylated in 23% of PDAC patients but in 0% of healthy patients (142).

Methylation of the promoter region of *GSTP1* has been analyzed in ctDNA samples. Using bisulfite treatment to maintain a marker for methylated cytosine residues, 50% of HCC patients from China have been observed exhibiting *GSTP1* promoter hypermethylation on ctDNA extracted from serum. However, this 50% incidence rate was also observed for patients with liver cirrhosis, suggesting a lack of specificity in ctDNA analysis (143).

In addition to analysis of promoter methylation, *GSTP1* has also proved beneficial in ctDNA analysis as an amplification locus for overall assessment of ctDNA levels. ctDNA has been shown to be significantly higher in HCV-HCC (141 ng/mL) patients than those in HCV carriers without HCC (34 ng/mL) and control patients (46 ng/mL) (144). The same group performed another study the next year with more HCV-induced HCC patients, demonstrating similar results for HCV-HCC (116 ng/mL) and HCV carriers (34 ng/mL). This increased ctDNA level was significantly associated with worse survival (145).

### Global Hypomethylation

The overall level of DNA methylation, in addition to specific oncogenic methylations on promoter regions, can also be a marker for cancer. Global DNA hypomethylation promotes genomic instability, and the methylation status of LINE-1 is often used as a marker for global DNA methylation. LINE-1 is a transposable element, i.e., a DNA sequence that moves and duplicates within the genome and makes up ~17% of the genome. Its hypomethylation, representative of global hypomethylation, is associated with a poor prognosis in many cancers (146). LINE-1 methylation levels have been shown to be decreased in HCC cases (146, 147), as well as pancreatic cancer cases (148), compared to healthy controls.

The methylation status of LINE-1 from ctDNA of HCC patients has been analyzed, showing that the percentage of unmethylated LINE-1 was significantly higher for HCC compared with healthy controls. Furthermore, LINE-1 hypomethylation could be correlated significantly with advanced tumor stages, indicating that LINE-1 hypomethylation is a significant and independent prognostic factor for overall survival (149).

### Gene Panels

Liver cancer is associated with many types of mutations at a moderate abundance, compared to the domination of the pancreatic cancer landscape by *KRAS*. ctDNA analysis methods have also used gene panels in order to improve sensitivity and specificity in both HCC and PDAC.

By looking at specific mutation hotspots in the genes *CTNNB1, TP53*, and the *TERT* promoter, a study identified mutations in 20% of patients. In addition, by quantifying total cell free DNA with a double stranded DNA stain similar to SYBR green, it was shown that total cell free DNA amount was not correlated with mutation status. Despite the limited promise of this study, a significant correlation between detectable mutation status and survival probability was observed (150). The same genes have been used in a panel in a ddPCR assay of ctDNA from HCC patients, with mutation detection at a higher incidence (56%) in this case (53). Furthermore, a deep sequencing technique for ctDNA, which amplified and analyzed 46 coding and non-coding genes, detected mutations in 63% of HCC patients (151).

Since aberrant methylation is a key part of the HCC mutational landscape, methylation marker panels have also been used for ctDNA analysis. In one study, a methylation marker panel was identified using a learning set of patients, identifying methylation in genes such as *NOTCH3* and *PPFIA1*. It was then tested on a different set of patients and showed a higher combined diagnosis score for HCC than healthy controls or liver disease. This panel was also a significant predictor of overall survival (152). Another study used a set of 4 methylation markers (*RGS10, ST8SIA6, RUNX2, VIM*) to detect HCC, with 94% sensitivity and 89% specificity (59). It should be noted that the methylation markers used in these studies are not methylation events which driver disease progression, such as *TERT* promoter methylation.

Panels of markers that analyse copy number variation have also been used for HCC detection. Using NGS to detect a panel of size alterations (e.g., gain in chromosomal region 1q and loss in chromosomal region 13q), HCC has been correctly identified in 84% of patients, with 100% specificity against cirrhosis and chronic hepatitis (63).

Though most combinatorial studies have analyzed HCC, some studies have evaluated ctDNA from PDAC with gene panels. One study analyzed the concentrations of mutated DNA for a multiple gene panels to test their diagnostic potential for PDAC identification. These panels were *KRAS* alone, *TP53* alone, *TP53* in combination with *SMAD4*, or a full panel of 9 genes including *KRAS, TP53, SMAD4* as well as other genes such as *CDKN2A*. For PDAC vs. control, the highest sensitivity (85.3%) was achieved by the 9 gene panel, whereas when comparing PDAC vs. intraductal papillary mucinous neoplasm (IPMN), *TP53* + *SMAD4* showed the highest sensitivity (64.7%). The *TP53* + *SMAD4* combination, as well as *TP53* alone, showed 100% specificity for PDAC vs. control, with the 9 gene panel less specific at 83.4%. *KRAS* was also highly specific at 91.7%. For PDAC vs. IPMN, the 9 gene panel was the most specific (85.7%) (80). This study highlights well that increasing the number of genes analyzed does not necessarily improve detection as both sensitivity and specificity must be considered.

## Conclusion and Future Insights

ctDNA analysis is emerging as a sensitive and specific method for analyzing the mutational landscape of patients with HCC and/or PDAC. Over the years, a multitude of ctDNA studies have identified the presence, or indeed absence, of mutations associated with either of these gastrointestinal diseases. ctDNA research has progressed by improving and updating detection and analysis techniques, and by understanding how to apply results from ctDNA analysis in a clinical setting.

A key goal of ctDNA research is for analysis of ctDNA from an individual patient to accurately represent the mutational landscape of that patient, and therefore be useful in diagnosing any malignancies such as PDAC or HCC, and dictating clinical practice (1). PDAC and HCC share many common driver mutations, e.g., alterations within the genes *TP53* and *CDKN2A*, but other frequent mutations only occur in one of these pathologies, e.g., *KRAS* mutations are abundant in PDAC but rare in HCC. Models for the mutational development of PDAC and HCC are well-established (Figure 2) and indicate how specific mutations drive steps along the pathway from health to disease, though heterogeneity in mutational profiles has limited current understanding.

**Figure 6:**
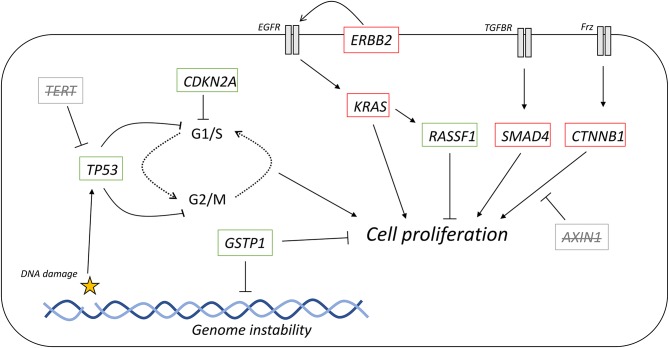
Representation of the tumor mutational landscape by ctDNA analysis. Commonly mutated genes in HCC and PDAC, and their role in cell signaling. Those genes with strikethrough have none or limited results in studies detecting these mutations. Red outline, commonly downregulated in tumors; green outline, commonly upregulated in tumors.

ctDNA mutational analysis represents the end of a long process that begins with the occurrence of somatic mutations within tumors cells. Inevitably, the rate at which specific mutations are then detected in ctDNA often differs from that detected in tumor biopsies. For example, mutations in *TP53* in PDAC are observed between 55 and 75% from tumor biopsies, but the range of incidence rates for ctDNA is from 11 to 59%. In this case, part of the reason could be that the ctDNA analysis only looks at the specific mutation R249S and does not consider others. However, the G12D mutation in *KRAS* in PDAC has been shown in tumors at an incidence of 98%, yet ctDNA analysis has detected this specific mutation at lower rates (33–94%). Additionally, it must be noted that many mutations often detected within the liver or pancreas have either been detected at a low incidence or not detected at all in ctDNA (Figure 6). For example, *TERT* promoter mutations are highly prevalent in HCC but have yet to be specifically detected in ctDNA from HCC patients, though have been used as part of a gene panel for ctDNA analysis (150).

The variance between the tumor and ctDNA mutational landscape is likely to be primarily generated from the variety of isolation and detection techniques and tumor heterogeneity, but other factors may also be involved. ctDNA only makes up part of the cell free DNA present within the body, and as such, non-mutated DNA fragments are also included in the analysis which dilutes ctDNA. Since ctDNA is often released through apoptosis, it can be speculated that the cells that more readily undergo apoptosis could be those where the driver mutation is not present, leading to less mutated DNA in the circulating population. Furthermore, it has been demonstrated that different DNA sequences exist at different concentrations within plasma, and it has been suggested that sequence may affect the rate of DNA cleavage within the blood (153). This may underlie why some genes are more readily detectable than others.

One key aim of ctDNA is to determine the genotype of the patient, which can be used to dictate therapy choices. For example the *BCR-ABL* oncogene, present in various leukaemias, can be targeted with the specific agent imatinib (154). Other genotypes are associated with a predicted lack of response to therapy e.g., mutant *KRAS* in colorectal cancer is associated with a lack of response to the therapeutic cetuximab (155). Some of the studies we highlight here link mutational status in ctDNA to survival metrics (e.g., 98, 107, 116, 124). Correlations between mutations, as detected by ctDNA analysis, and therapy response to specific agents are required for combining the field of ctDNA mutational analysis with clinical prognosis and therapy choices.

The diagnostic and/or prognostic potential of a particular mutation requires high sensitivity and specificity, and therefore high accuracy. Many of the driver mutations that have been so far detected through ctDNA show limited accuracy, though others show more promise in reaching the goal of 100% accuracy. The hypothetical perfect analysis technique would be able to detect a mutational change or changes present in all cases of the disease (i.e., 100% sensitive) and in no cases without the disease (i.e., 100% specific). An improved understanding of the molecular biology that drives disease initiation will be informative for identifying all possible mechanisms for the disease. For example, while *KRAS* mutations are highly prevalent in early PDAC development and seen as driver mutations, they are not present in 100% of cases (156). An understanding of which mutations drive these *KRAS*-independent developments may converge on a pathway, or set of pathways, from which all known disease progressions develop from. A full ctDNA analysis of the genes encoding these proteins may show a perfect sensitivity and specificity.

Sensitivity and specificity are highly dependent on many pre-analytical parameters, e.g., plasma separation and method of ctDNA isolation, and optimization of the assay may require extra steps within the workflow (65). However, perfect sensitivity and specificity may not necessarily be required for therapy choices based on mutation status. If known ctDNA mutations are significantly associated with various survival metrics, including post-therapy survival, then therapeutic decisions could be made. A patient may still have the disease state, and further monitoring may be required, but if the patient has a form of the disease that does not present with ctDNA mutations, their survival prospects may be better and hence therapy would not be needed. A highly sensitive, but perhaps less specific, assay could be used for longitudinal monitoring purposes, to ensure that patients are not characterized as healthy when they have the disease.

Both HCC and PDAC develop through multiple stages, and these stages are associated with specific mutations e.g., *KRAS* as an early mutation that occurs in PanIN, and *TERT* promoter methylation that occurs following liver cirrhosis. As such, more studies are needed which analyse ctDNA during different parts of disease progression. One good example of ctDNA monitoring is a study which analyzed hypermethylation of the *GSTP1* promoter hypermethylation in healthy individuals and patients with cirrhosis and HCC (141). Since there is sometimes a discrepancy between tumor mutation status and ctDNA mutation status, more studies that specifically analyse ctDNA mutations at different tumor progression stages will improve our understanding of the circulating mutational landscape, a landscape which is clinically available.

One feature of diagnosis rarely considered within studies that analyse ctDNA is the differentiation between different diseases. For example, many studies report high specificity for HCC compared to healthy and other liver disease states, but do not compare HCC vs. diseases of other organs, such as PDAC. *KRAS* is seen as a high accuracy marker for PDAC, though has also been detected in the ctDNA of colorectal cancer patients (157). Similarly, *TERT* promoter mutations, seen as key driver mutations in HCC, are present in multiple other cancers including bladder and skin cancer (158). Gene panels, which assess multiple genes, may be able to differentiate cancers from each other, if further detail on the cancer mutational landscape, as present in ctDNA, could be found. Studies that compare and analyse ctDNA from a cohort of patients exhibiting various diseases may lead to identification of gene panels that show high specificity. These efforts would likely be affected by intertumor heterogeneity amongst patients. Furthermore, choice of bodily fluid, e.g., pancreatic juice instead of blood, may allow the specific identification of particular cancers.

In conclusion, the ctDNA mutational landscape differs from the tumor mutational landscape, and research must be undertaken to evaluate the mechanisms behind this discrepancy. Future studies should also, if possible, report on how ctDNA mutation detection is related to survival metrics and/or therapy response. With this information, ctDNA analysis may become an indispensable tool in analyzing, and basing therapeutic decisions on, the mutational status of tumors in individual patients, and further progression in the field of personalized therapy.

## Author Contributions

AR and ARH wrote the manuscript.

### Conflict of Interest

The authors declare that the research was conducted in the absence of any commercial or financial relationships that could be construed as a potential conflict of interest.
